# Editorial: Environmental risk factors and inflammation in psychiatric and neurological disease

**DOI:** 10.3389/fpsyt.2022.1080761

**Published:** 2022-12-07

**Authors:** Ute-Christiane Meier, Sreeram Ramagopalan, Norbert Müller

**Affiliations:** ^1^Institut für Laboratoriumsmedizin, Klinikum der Universität München, Munich, Germany; ^2^Department of Psychological Medicine, Institute of Psychiatry, Psychology and Neuroscience, King's College London, London, United Kingdom; ^3^Global Access, F. Hoffmann-La Roche, Basel, Switzerland; ^4^Department of Psychiatry and Psychotherapy, Ludwig-Maximilians Universitaet München, Munich, Germany

**Keywords:** neurological disease, neuropsychiatric diseases, environmental risk factors, inflammation, immune system, neuroinflammation

Neuropsychiatric and neurological diseases are multifactorial entities. Genetic susceptibility plays an important role as do environmental risk factors. In contrast to the genetic susceptibility, environmental risk factors, if identifiable, may offer attractive strategies for intervention and warrant further investigation. There is strong evidence for a role of environmental risk factors such as viral infections (e.g., Epstein-Barr virus) and vitamin D deficiency and ensuing neuroinflammation in multiple sclerosis (1–3). Emerging evidence highlights that the immune system may also play an important role in the pathogenesis of psychiatric disorders. Risk factors for multiple psychiatric disorders comprise the exposure to environmental triggers that stimulate an immune response, such as childhood and adult infections as well as *in-utero* or parental infections, childhood adversity, and acute or chronic stress.

This was highlighted by recent findings, which showed that genetic variants associated with psychiatric disorders were enriched at epigenetically active sites in lymphoid cells especially T- and memory B-cells (4). The observed abnormalities in the resolution of immune responses to environmental risk factors may potentially lead to chronic, low-grade peripheral inflammation. These important findings may pave the way for studies to improve treatment and prevention strategies, as well as for the development of novel inflammation platforms to detect and measure low-grade peripheral inflammation. Altered immune responses with increased C-reactive protein (CRP) levels, increased pro-inflammatory cytokines, inflammasome activation and elevated white blood cell counts have been observed in case-control studies of many psychiatric disorders, including schizophrenia, major depressive disorder (MDD), bipolar disorder, Alzheimer's disease, autism spectrum disorder (ASD) and attention deficit hyperactivity disorder (ADHD) (4).

This Research Topic brings together eight articles, both reviews and original articles, which highlight recent advances in the field. The first two article focus on ASD and inflammation.

The review by Whiteley et al. summarizes the emerging evidence of a connection between autoimmune encephalitis (AE) and ASD. The authors put forward the idea that where autism is accompanied by regression and atypical onset patterns, it may be prudent to investigate whether a differential diagnosis of AE would be more appropriate. AE comprises a group of conditions characterized by the body's immune system mounting an attack on healthy brain cells causing brain inflammation. The resultant cognitive, psychiatric and neurological symptoms that follow AE have also included ASD or autism-like traits and states.

The original research study by Wang et al. sheds light on how neuroinflammation may contribute to the pathogenesis of ASD and pave the way for the development of anti-inflammatory therapeutic strategies for ASD by understanding mechanistic pathways. The authors reported elevated plasma levels of the proinflammatory chemokine ligand 5 (CCL5) in children with ASD. The abnormal activation of mTOR signaling in ASD was shown to be associated with elevated CCL5 gene expression. *In-vitro* studies showed that the suppression of mTOR reduced the gene expression and release of CCL5 from human microglia. The authors suggested that targeting mTOR can provide a potential therapeutic strategy for ASD. Understanding of core signaling pathways may help take an important step from discovering autism-specific mutations and their functional effects to aberrant signaling pathways in ASD. Although there is currently no effective pharmacological cure for ASD, the use of anti-inflammatory agents has shown to prevent social deficits in autism animal models. Anti-inflammatory dietary interventions have also been reported to improve attention and sociability defects in children with ASD.

The two following articles focus on the role of neuroinflammation in Alzheimer's disease, which is an active area of research and warrants further study. Neuroinflammation is thought to drive neurodegeneration in Alzheimer's disease pathogenesis, thus there is a drive to develop novel therapeutic strategies. Neuroimaging and biomarker studies have reported that an inflammatory response is present within the brain and in the periphery in mild cognitive impairment (MCI) conditions and increases with disease progression. There is an urgent need to develop novel therapeutic compounds, which modulate these processes.

Kim et al. studied the effect of Sorafenib on neuroinflammation *in-vitro* and *in-vivo*. Sorafenib is FDA- approved for the treatment of primary kidney and liver cancer. Sorafenib may also have therapeutic potential for suppressing neuroinflammatory responses in the brain as it impacted *in-vitro* on LPS-mediated neuroinflammatory response in microglial cells and was found to reduce COX-2 induction in microglial cells and primary astrocytes. *In-vivo* administration of Sorafenib suppressed microglial/astroglial kinetics and morphological changes and COX-2 mRNA levels by decreasing AKT phosphorylation in the brain. In an Alzheimer's disease model (FAD mice), Sorafenib treatment reduced astrogliosis and may therefore have therapeutic potential for suppressing neuroinflammatory responses in the brain.

Toppi et al. present data on homotaurine, a potential therapeutic compound for the treatment of Alzheimer's disease due to its neuroprotective and anti-inflammatory activities. Homotaurine is a natural amino acid first identified in different species of marine red algae. The authors reported the modulation of serum cytokine levels after homotaurine treatment in patients with mild cognitive impairment (MCI) with decreased circulating levels of the pro-inflammatory cytokine IL-18 and up-regulation of the anti-inflammatory cytokines IL-10 and IL-33. There was a correlation between serum IL-33 and IL-10 levels and episodic memory performance scored in MCI patients.

Inflammation also plays an important role in MDD. The mini-review by He et al. summarizes the current knowledge on white matter changes as characteristic feature of MDD and highlights that inflammatory factors play a critical role in the onset and progression of MDD. Depressive disorder has been linked to these changes, which could be caused by a combination of genes and environmental factors, neuroinflammation, impaired cerebral blood flow and impaired blood-brain barrier function. Neuroinflammation is known to impact on the formation and integrity of the myelin sheath, ultimately resulting in white matter damage. As white matter changes in MDD patients were related to differential diagnosis, the severity of MDD, treatment effect evaluation and prognosis, the authors suggested that imaging methods should be developed and used to provide patients with individualized treatment plans to enhance treatment effect and prognosis.

The Research Topic also comprises two articles on stroke and assesses the role of inflammation in this condition.

Qu et al. studied the association between circulating TSG-6 and stroke in humans. They reported that plasma concentration of tumor necrosis factor-stimulated gene 6 (TSG-6) may act as a novel diagnostic and 3-month prognostic indictor in non-cardioembolic acute ischemic stroke (AIS). TSG-6 is a multifunctional secretory protein exhibiting anti-inflammatory and tissue protective properties. The study showed that TSG-6 and interleukin-8 (IL-8) may act as valuable biomarkers for assessing 3-month outcome and highlighted that further research into the relationship between TSG-6 and ischemic stroke was warranted.

The interplay between gastrointestinal dysfunction and systemic inflammation is the focus of intense research. It is increasingly recognized that intestinal pathological changes are correlated to Parkinson's disease, multiple sclerosis as well as Alzheimer's disease. Animal research also showed that systemic inflammation could increase the risk of stroke and is associated with less favorable clinical outcome.

The original article by Zheng et al. studied gut microbiota changes and systemic inflammation in cryptogenic stroke patients and reported that gut dysbiosis was associated with the severity of cryptogenic stroke and enhanced systemic inflammatory response. There was an association between gut inflammation and enhanced systemic inflammatory responses in these patients. The gut microbiota differed between cryptogenic stroke patients and normal controls and gut dysbiosis was related to systemic inflammation and the stroke severity and infarct volumes.

Lei et al. reported that gut microbiota-mediated metabolic restructuring aggregated emotional deficits after anesthesia/surgery in rats with preoperative stress. The study shed light on the relationship between metabolite, gut microbiota and neuroinflammation after anesthesia/surgery in rats with preoperative stress. The study showed that abnormalities in the gut microbiota contributed to postoperative metabolic restructuring, neuroinflammation and psychiatric deficits in susceptible individuals.

In conclusion, this Research Topic presents a selection of articles that underscore the importance of research on environmental risk factors, low-grade peripheral inflammation and neuroinflammation in neuropsychiatric and neurological disease. The perspective arisen from these articles may contribute to novel interventions and treatment strategies as well as the development of innovative platforms to measure low-grade peripheral inflammation.

## Author contributions

All authors listed have made a substantial, direct, and intellectual contribution to the work and approved it for publication.

## Conflict of interest

Author U-CM holds patents EP3011344B1 and US11346849. Author SR was employed by F. Hoffmann-La Roche. The remaining author declares that the research was conducted in the absence of any commercial or financial relationships that could be construed as a potential conflict of interest.

## Publisher's note

All claims expressed in this article are solely those of the authors and do not necessarily represent those of their affiliated organizations, or those of the publisher, the editors and the reviewers. Any product that may be evaluated in this article, or claim that may be made by its manufacturer, is not guaranteed or endorsed by the publisher.
